# Supplementary material for: Sub-nanometer confinement enables facile condensation of gas electrolyte for low-temperature batteries
Source: Nat Commun. 2021 Jun 7;12:3395. doi: 10.1038/s41467-021-23603-0 (PMC8184934; doi:10.1038/s41467-021-23603-0)
Supplement: Supplementary file 1 — Supplementary Information [file 41467_2021_23603_MOESM1_ESM.pdf]

Supplementary Information

Sub-Nanometer Confinement Enables Facile Condensation of Gas Electrolyte for Low-Temperature Batteries

Cai *et al.*

Supplementary Figures and Tables.

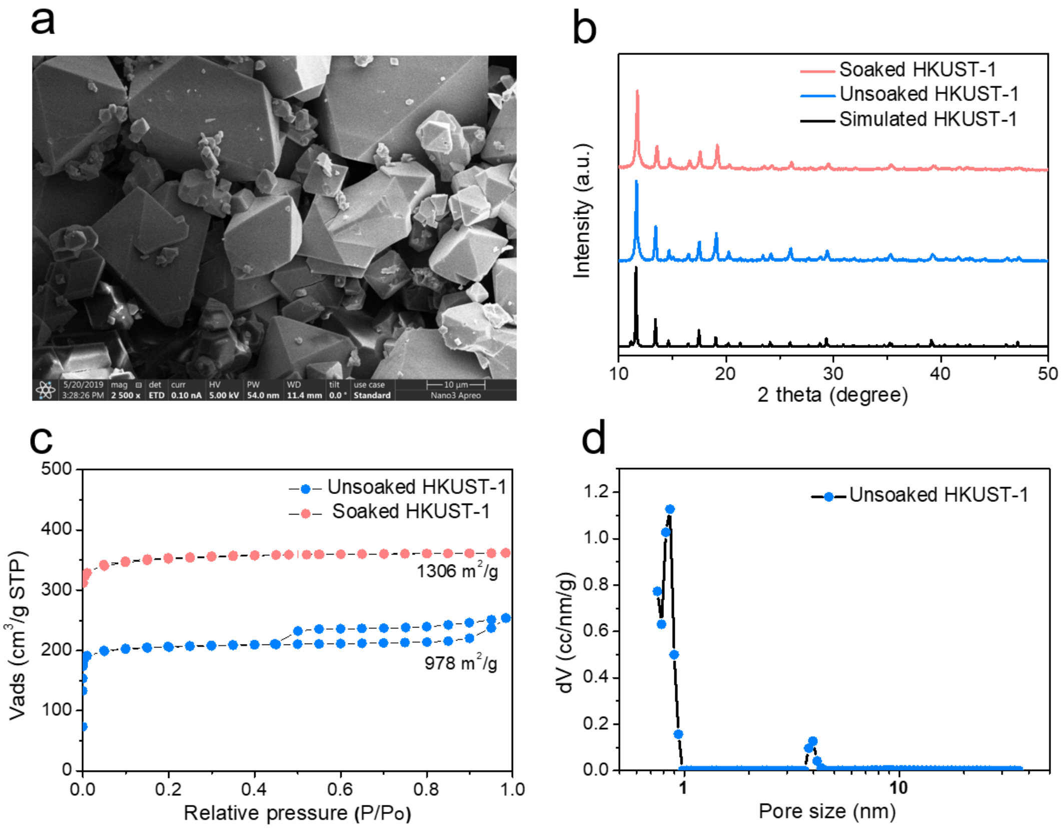

Supplementary Figure 1 | KUUST-1. a, SEM images of HKUST-1. XRD patterns (**b**), N_2 sorption isotherms (**c**), and pore size distribution profiles (**d**) of HKUST-1 before and after soaking in liquified FM.

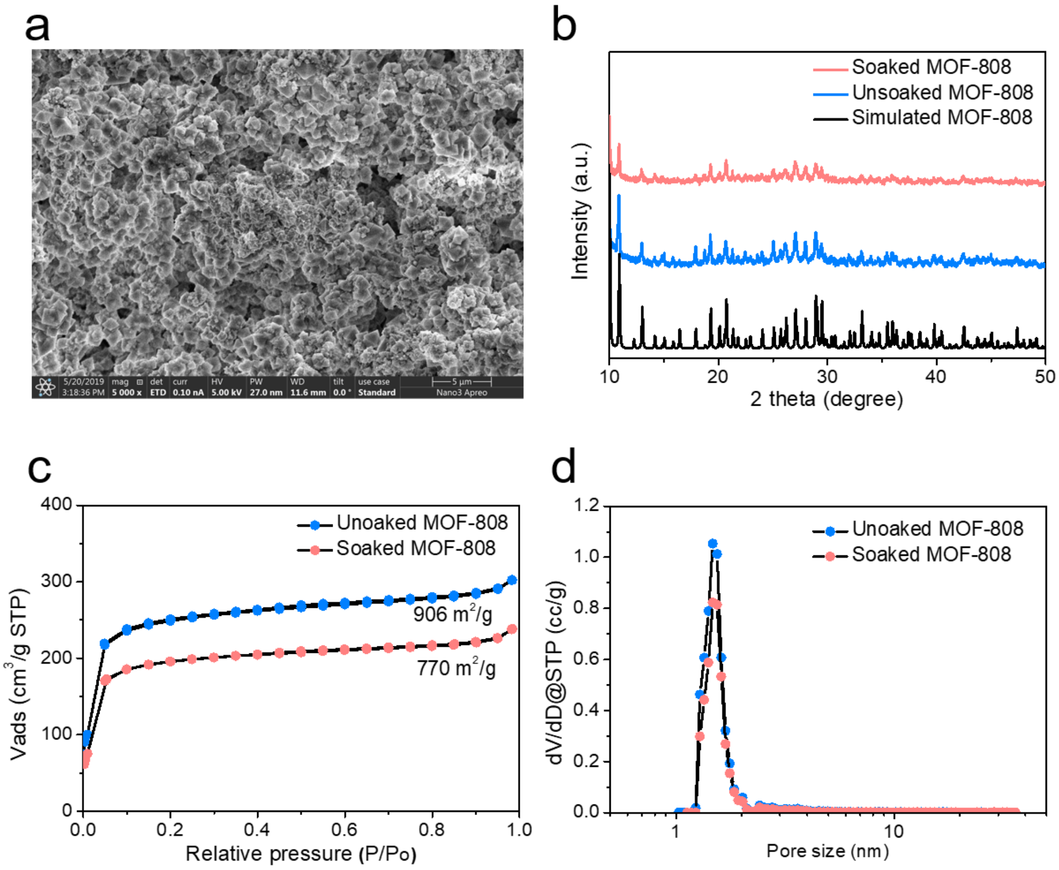

Supplementary Figure 2 | MOF-808. a, SEM images of MOF-808. XRD patterns (**b**), N_2 sorption isotherms (**c**), and pore size distribution profiles (**d**) of MOF-808 before and after soaking in liquified FM.

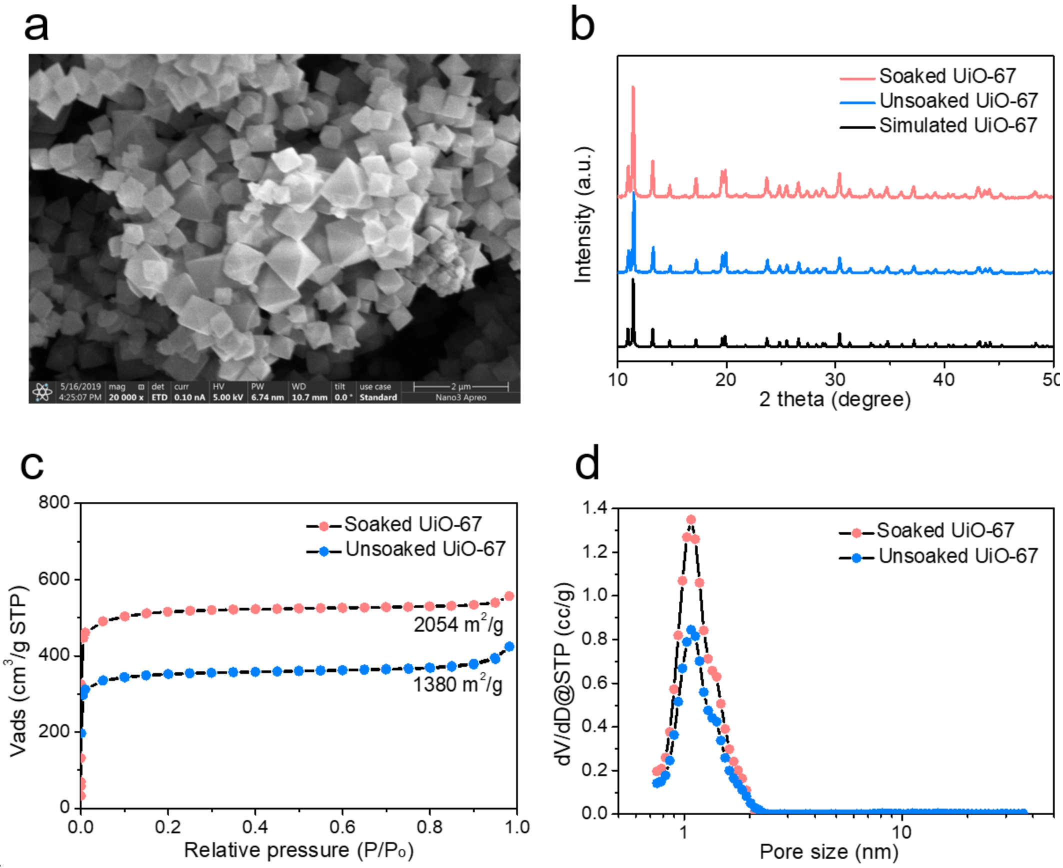

Supplementary Figure 3 | UiO-67. a, SEM images of UiO-67. XRD patterns (**b**), N_2 sorption isotherms (**c**), and pore size distribution profiles (**d**) of UiO-67 before and after soaking in liquified FM.

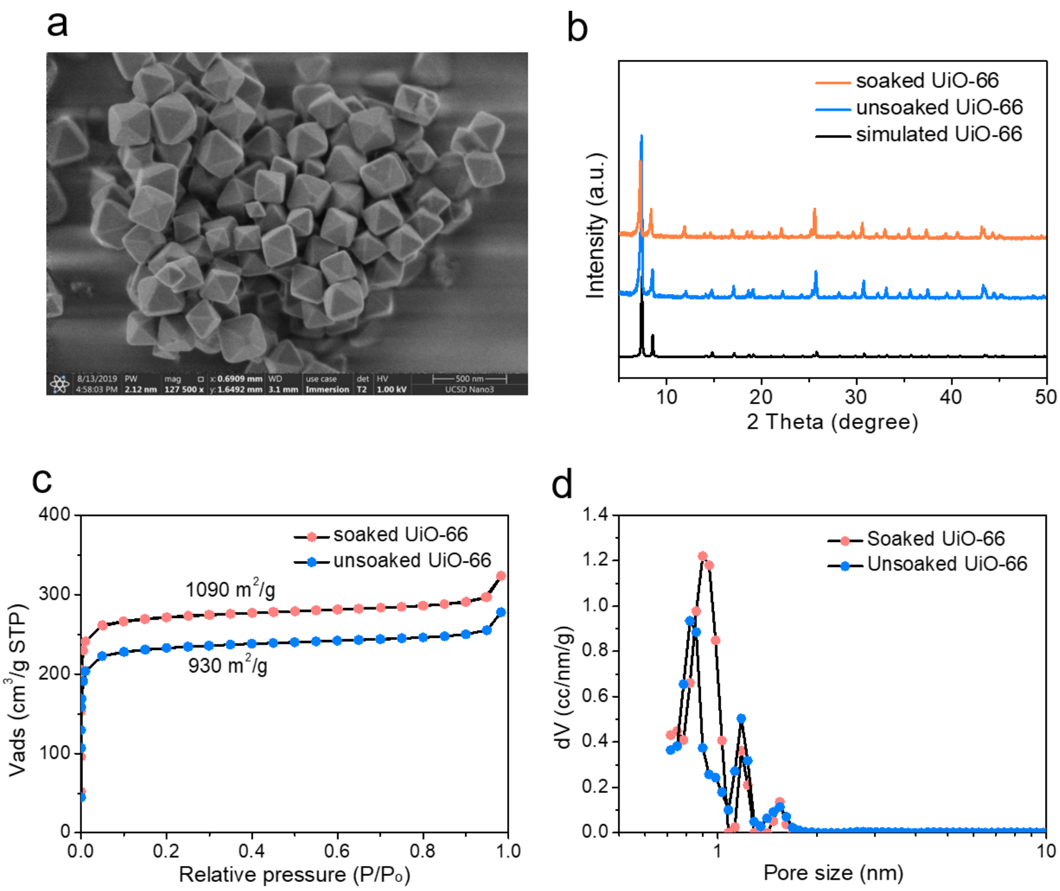

Supplementary Figure 4 | UiO-66. a, SEM images of UiO-66. XRD patterns (**b**), N_2 sorption isotherms (**c**), and pore size distribution profiles (**d**) of UiO-66 before and after soaked in liquified FM.

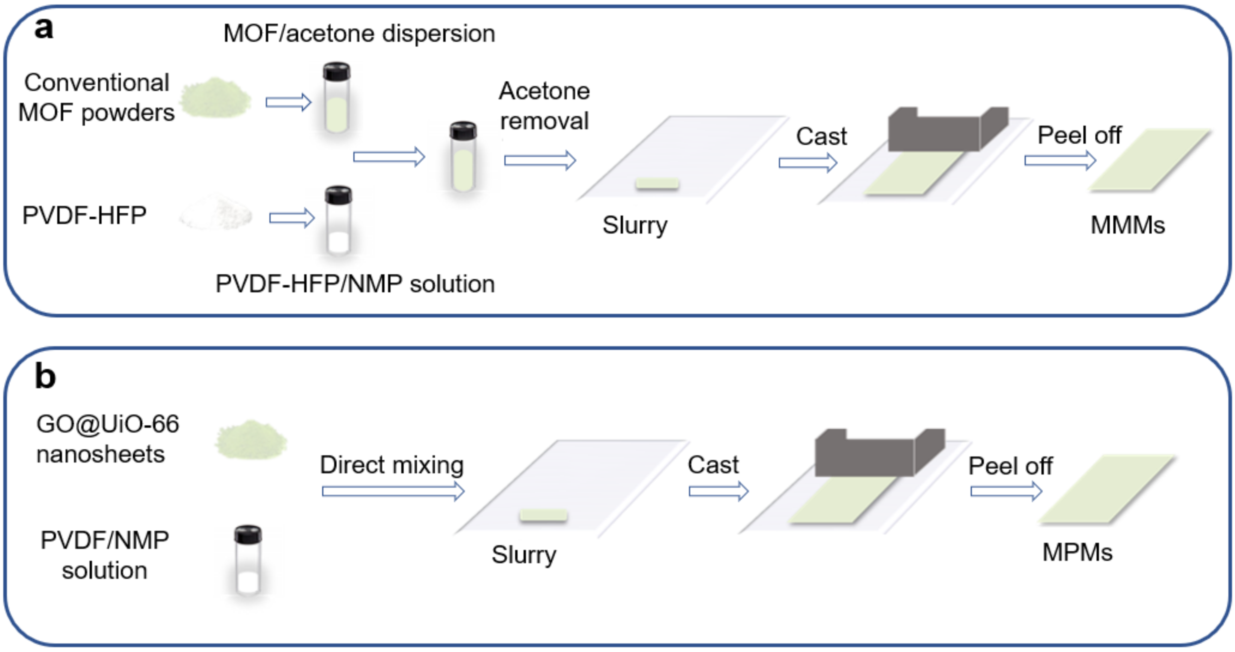

Supplementary Figure 5 | Illustration of the fabrication process of free-standing membranes. **a**, MOF powders-based MMMs. **b**, 2D GO@UiO-66-based MPMs.

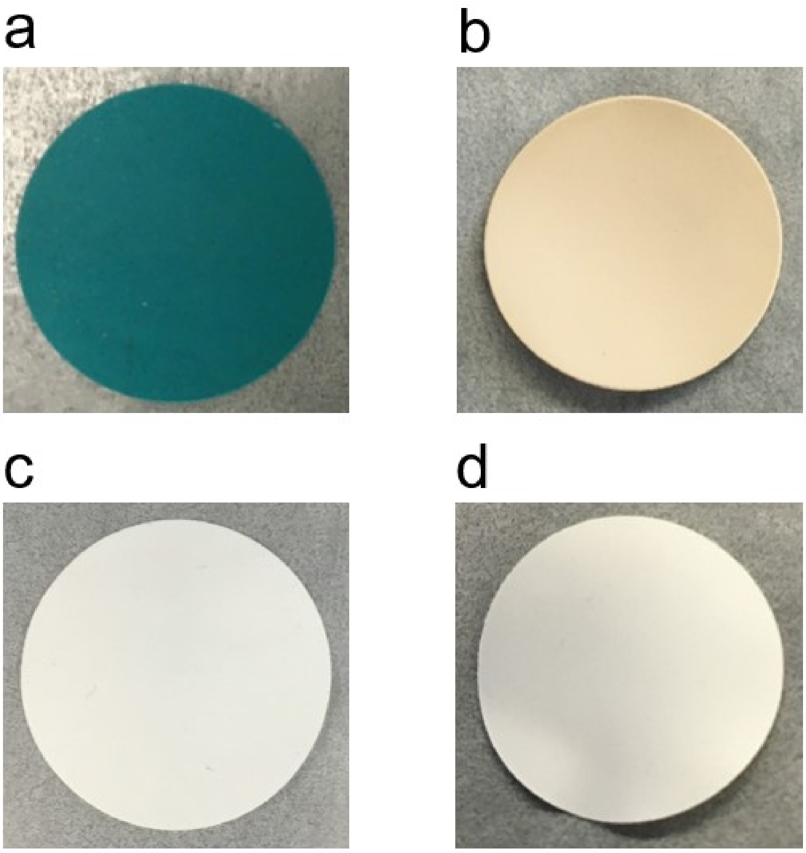

Supplementary Figure 6 | Photos of MMMs (Diameter: 3/8 inch; thickness: ~ 100 μm). MMMs with various MOFs: (**a**) HKUST-1, (**b**) MOF-808, (**c**) UiO-67, and (**d**) UiO-66.

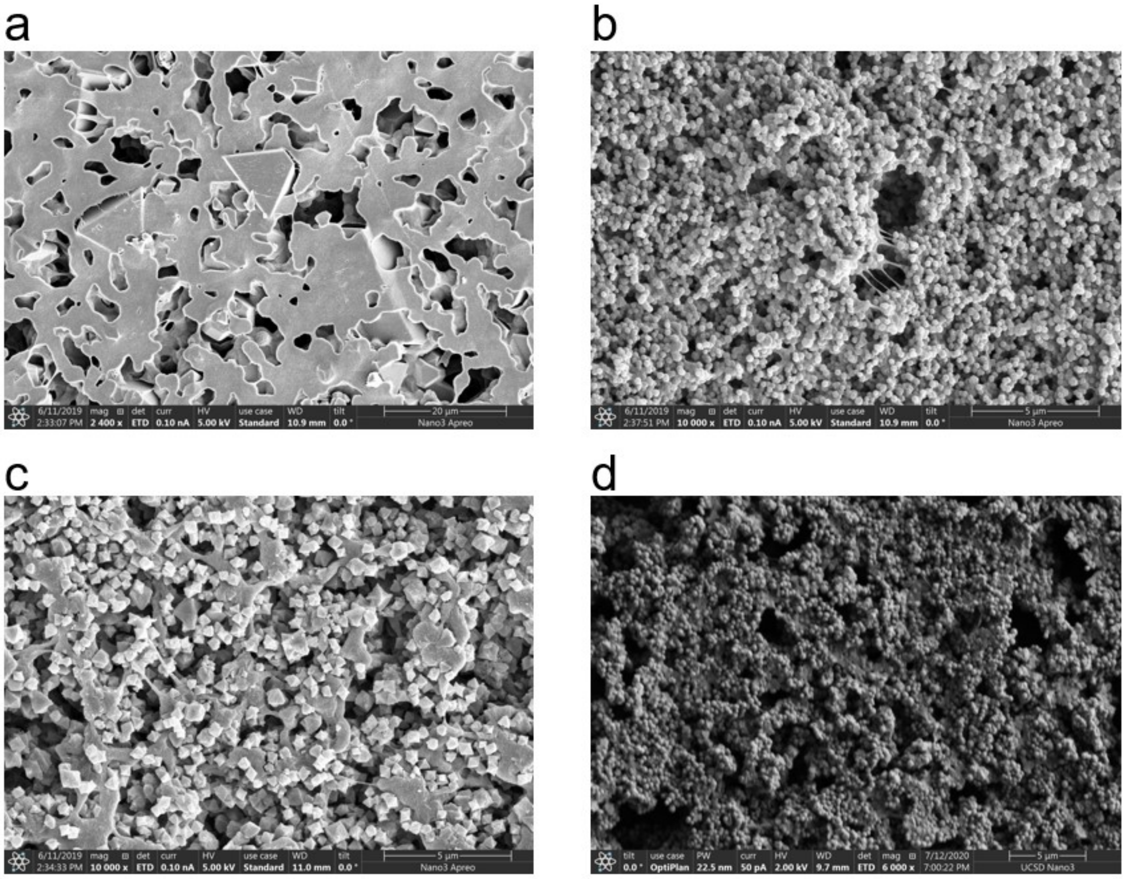

Supplementary Figure 7 | SEM images of MMMs. MMMs (Diameter: 3/8 inch; thickness: ~ 100 μm) with various MOFs: (**a**) HKUST-1, (**b**) MOF-808, (**c**) UiO-67, or (**d**) UiO-66.

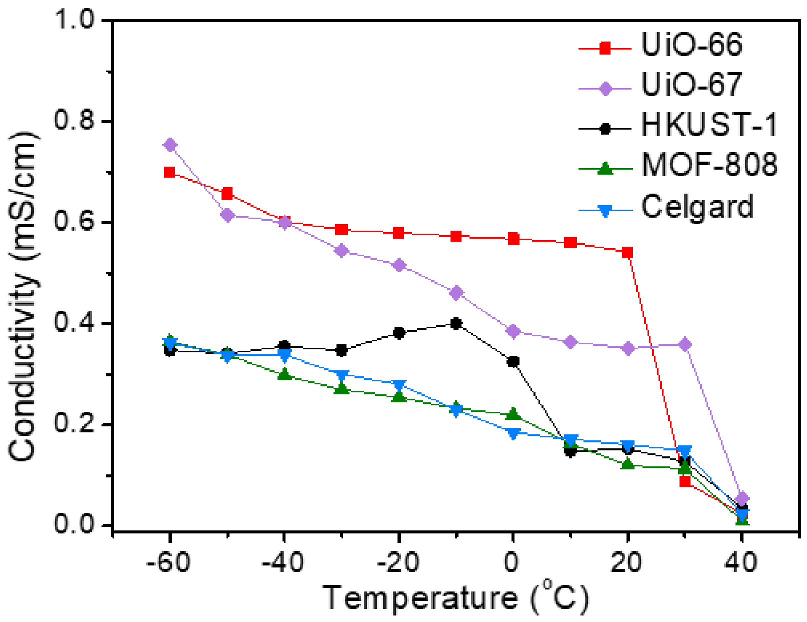

Supplementary Figure 8 | Ionic conductivity of FM-based electrolytes with various MMMs or Celgard membrane. Ionic conductivity of 0.3 M THF + 0.3 M LiTFSI in FM measured with various MMMs and commercial Celgard membrane at different temperatures, where two symmetric stainless-steel current collectors were set constantly at 500 μm for all of the measurements.

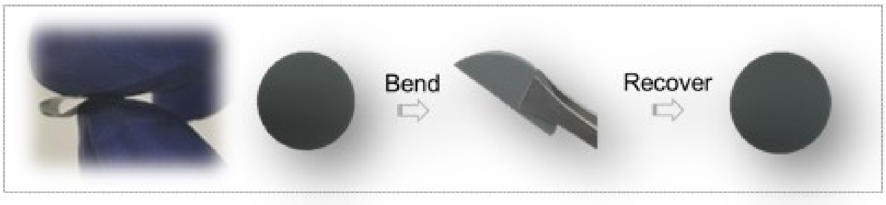

Supplementary Figure 9 | Photographs of MPM bending and recovery.

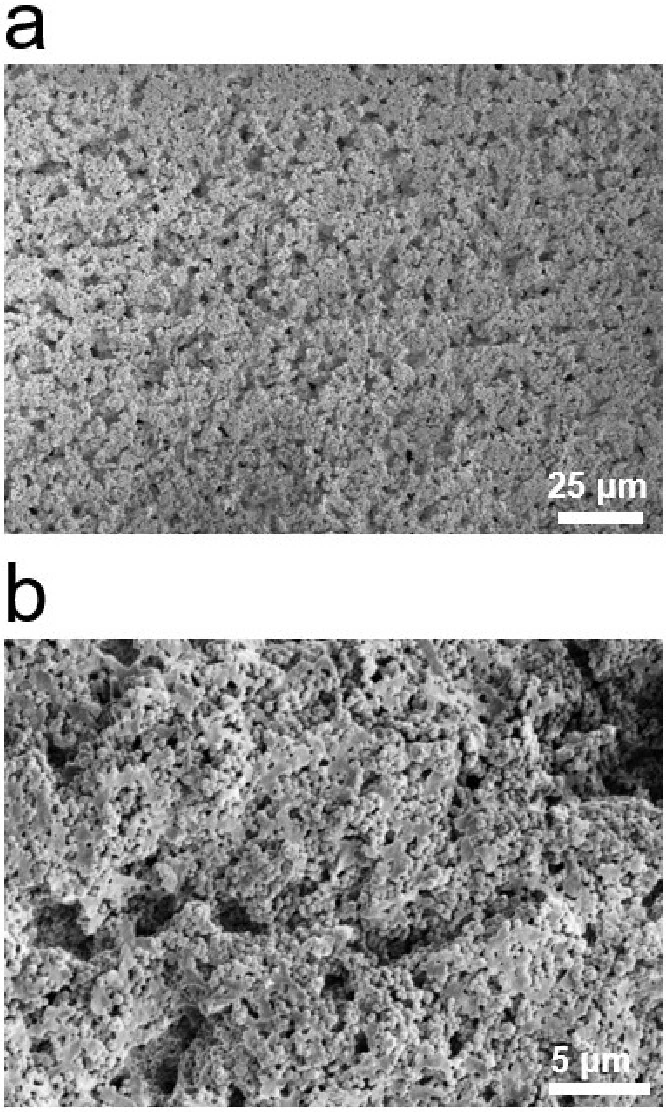

Supplementary Figure 10 | SEM images of 3D UiO-66 particle-based MMMs after soaking into 0.3 M LiTFSI in FM (room temperature, vapor pressure).

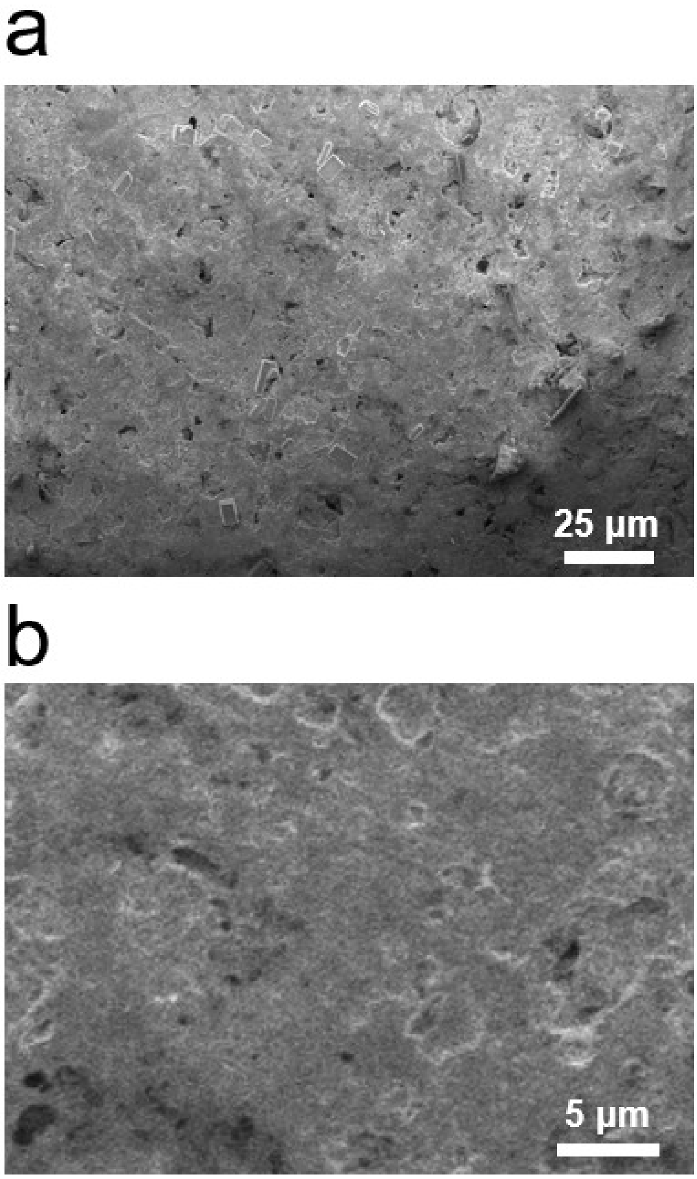

Supplementary Figure 11 | SEM images of 2D GO@UiO-66-based MPM after soaking into 0.3 M LiTFSI in FM (room temperature, vapor pressure).

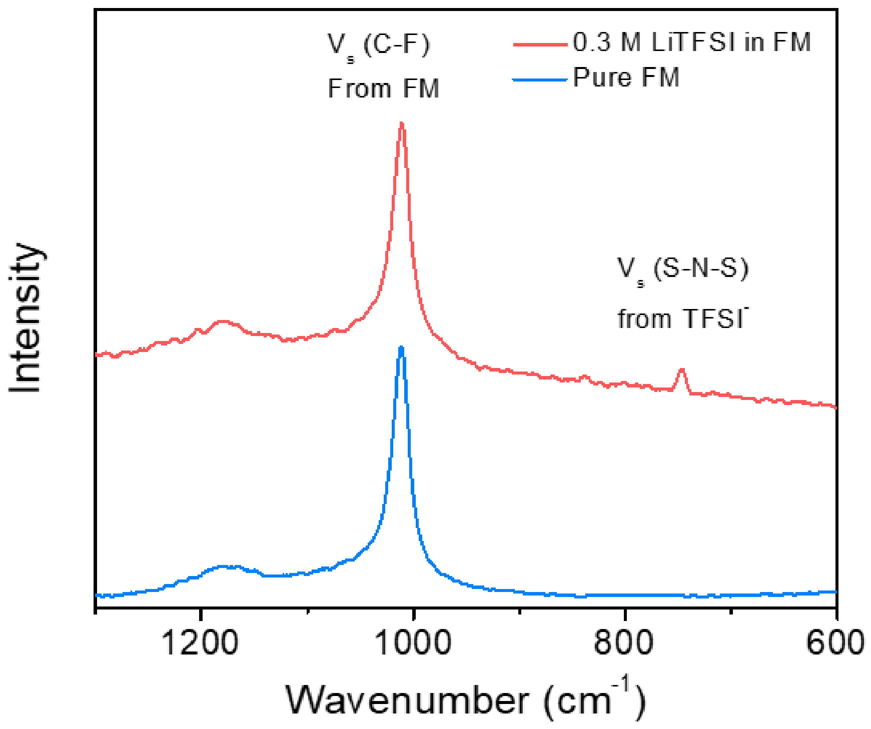

Supplementary Figure 12 | Raman spectra of pure FM and 0.3 M LiTFSI in FM.

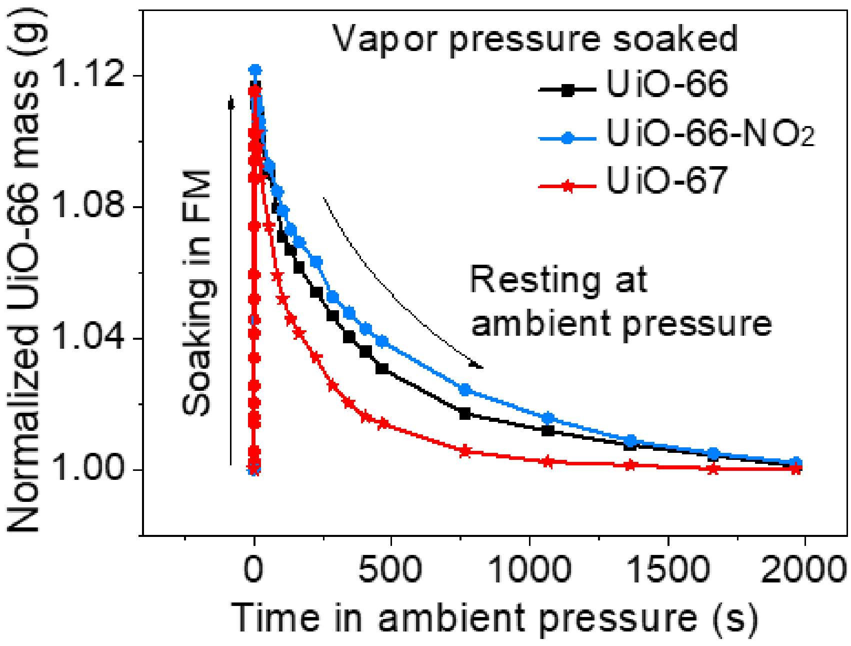

Supplementary Figure 13 | Mass change tests of liquified FM soaked UiO-66 and its analogues.

Supplementary Table 1 | Simulation parameters applied in MD/GCMC computations.

The pair interaction parameters and intramolecular parameters of UiO-66 were taken from published works.[1,2] FM molecule structure was optimized to obtain intermolecular/intramolecular parameters based on QM calculations at the MP2/aug-cc-pVTZ level of theory, with the partial atom charges taken from a published work.[3] CO_2 and CH_4 were described by the TraPPE-EH and TraPPE-UA forcefields respectively.[4]

Materials	Types	Equations	Parameters	
FM	Pair interaction	Lennard-Jones	C	σ = 3.304; ε = 0.0980; M = 12.01
			H	σ = 2.385; ε = 0.0456; M =1.01
			F	σ = 2.671; ε = 0.1165; M = 19.00
	Bond	Rigid (GCMC)<br>Harmonic (MD)	C-F	Rigid: r_0 = 1.389<br>Harmonic: r_0 = 1.389; K = 368
			C-H	Rigid: r_0 = 1.087<br>Harmonic: r_0 = 1.087; K = 333.5
	Angle	Rigid (GCMC)<br>Harmonic (MD)	H-C-H	Rigid: θ_0 = 110.3<br>Harmonic: θ_0 = 110.3; K = 34.79
			H-C-F	Rigid: θ_0 = 108.6<br>Harmonic: θ_0 = 108.6; K = 40.00
	Charges[3]		C	-0.2469
			F	-0.1950
			H	+0.1473
FM/UiO-66	Pair interaction	Lennard-Jones	C(FM)-Zr1(UiO-66)<br>F(FM)-Zr1(UiO-66)<br>H(FM)-Zr1(UiO-66)	σ = 3.485; ε = 0.0535<br>σ = 3.134; ε = 0.0583<br>σ = 2.961; ε = 0.0365
			C(FM)-O1(UiO-66)<br>F(FM)-O1(UiO-66)<br>H(FM)-O1(UiO-66)	σ = 3.498; ε = 0.0673<br>σ = 3.145; ε = 0.0734<br>σ = 2.972; ε = 0.0459

			C(FM)-C25(UiO-66)<br>F(FM)-C25(UiO-66)<br>H(FM)-C25(UiO-66)	σ = 3.966; ε = 0.0503<br>σ = 3.566; ε = 0.0548<br>σ = 3.370; ε = 0.0343
			C(FM)-O29(UiO-66)<br>F(FM)-O29(UiO-66)<br>H(FM)-O29(UiO-66)	σ = 3.498; ε = 0.0673<br>σ = 3.145; ε = 0.0734<br>σ = 2.972; ε = 0.0459
			C(FM)-O25(UiO-66)<br>F(FM)-O25(UiO-66)<br>H(FM)-O25(UiO-66)	σ = 3.633; ε = 0.0875<br>σ = 3.266; ε = 0.0954<br>σ = 3.086; ε = 0.0597
			C(FM)-C1(UiO-66)<br>F(FM)-C1(UiO-66)<br>H(FM)-C1(UiO-66)	σ = 4.128; ε = 0.0581<br>σ = 3.712; ε = 0.0633<br>σ = 3.507; ε = 0.0396
			C(FM)-C13(UiO-66)<br>F(FM)-C13(UiO-66)<br>H(FM)-C13(UiO-66)	σ = 4.118; ε = 0.0416<br>σ = 3.702; ε = 0.0453<br>σ = 3.498; ε = 0.0284
			C(FM)-H1(UiO-66)<br>F(FM)-H1(UiO-66)<br>H(FM)-H1(UiO-66)	σ = 3.211; ε = 0.0458<br>σ = 2.887; ε = 0.0499<br>σ = 2.728; ε = 0.0312
			C(FM)-H25(UiO-66)<br>F(FM)-H25(UiO-66)<br>H(FM)-H25(UiO-66)	σ = 0.0; ε = 0.0<br>σ = 0.0; ε = 0.0<br>σ = 0.0; ε = 0.0
The units of energy, distance, angle, mass and charge are kcal/mol, Angstrom, degree, g/mol and electron charge, respectively. Lennard-Jones equation is $E = 4\varepsilon[(\sigma/r)^{12}-(\sigma/r)^{6}]$. Harmonic equations are $E = K(r-r_0)^2$ (for bond) and $E = K(\theta- \theta_0)^2$ (for angle).				

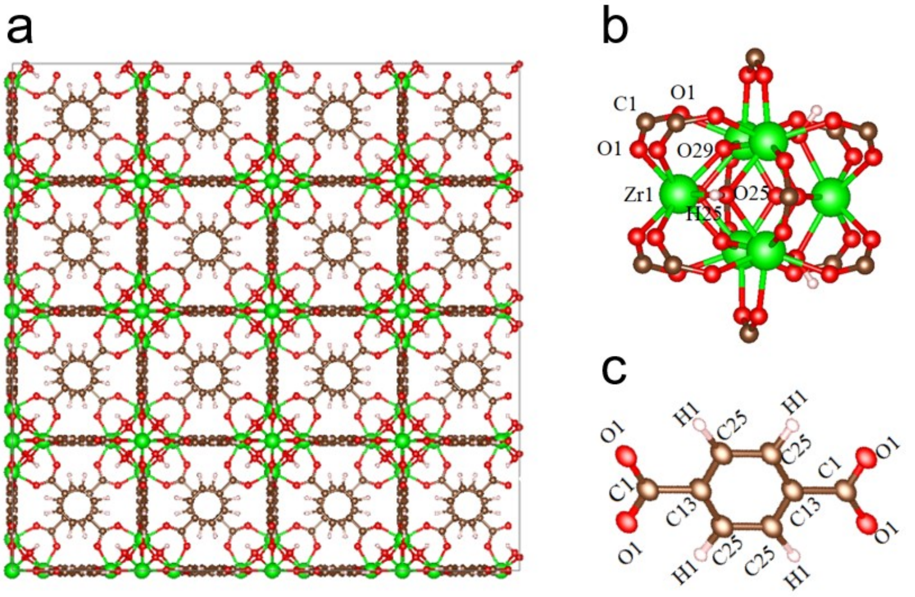

Supplementary Figure 14 | Simulation structure of UiO-66. The simulation structure for (**a**) UiO-66, compositing of (**b**) [$Zr_6O_4(OH)_4$] clusters and (**c**) BDC linkers.

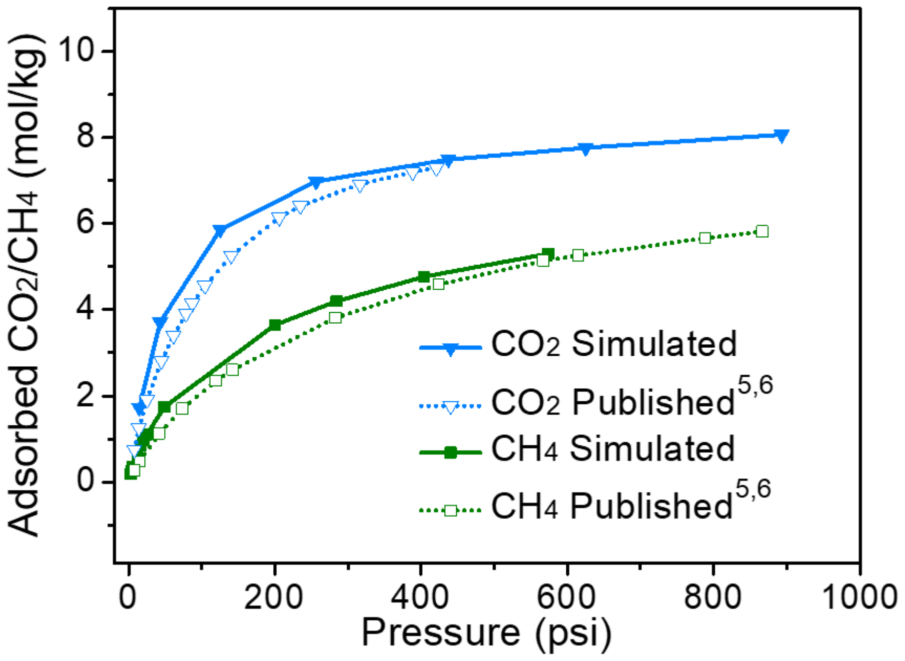

Supplementary Figure 15 | Simulated adsorption isotherms of UiO-66 confined CH_4 and CO_2 at room temperature, compared to other published data.[5,6] We find overall excellent agreement with published data, validating our current simulation approach.

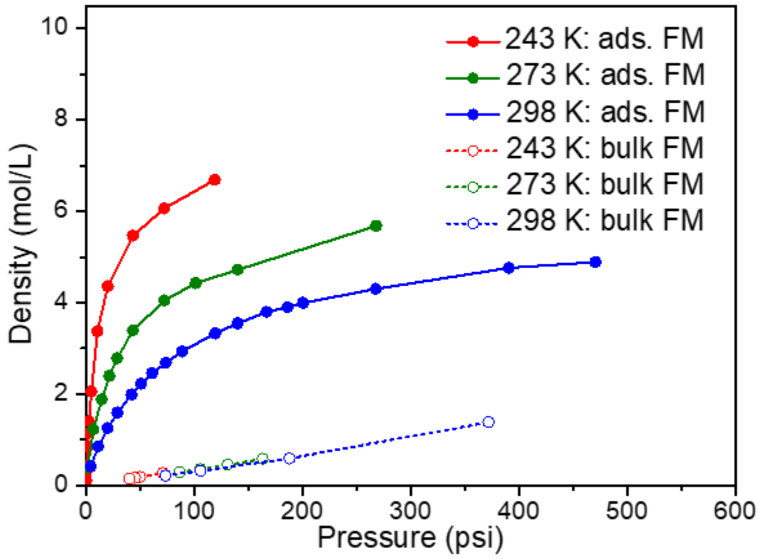

Supplementary Figure 16 | The densities comparison between adsorbed FM inside UiO-66 and free FM in bulk FM systems. The solid curves represent the FM densities inside UiO-66 and the dashed curves indicate the bulk FM densities, at the stated temperatures.

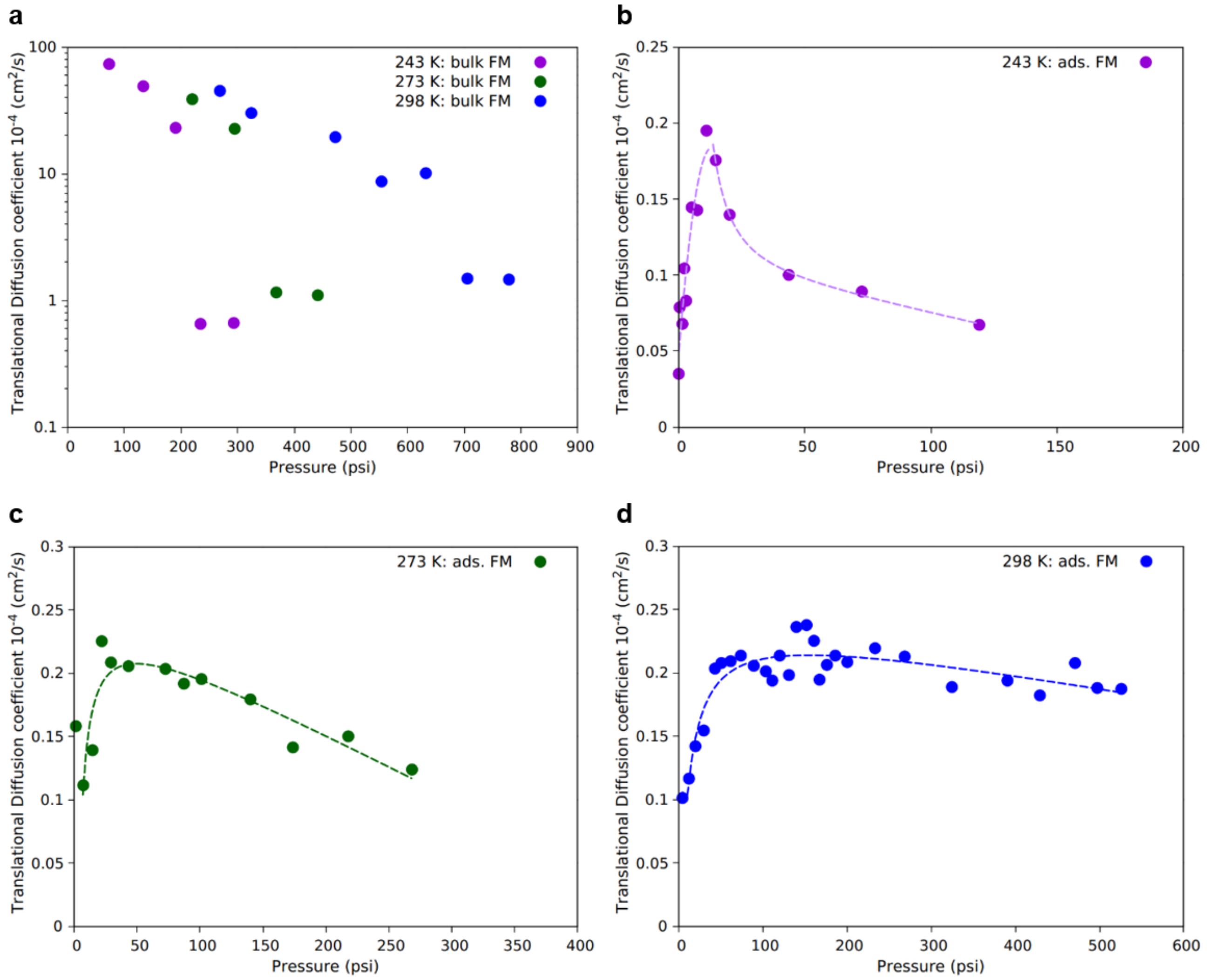

Supplementary Figure 17 | Simulated translational diffusion coefficients. Simulated translational diffusion coefficients of (**a**) bulk FM, and (**b**, **c**, **d**) adsorbed FM (ads. FM) in UiO-66 at different temperatures and pressures.

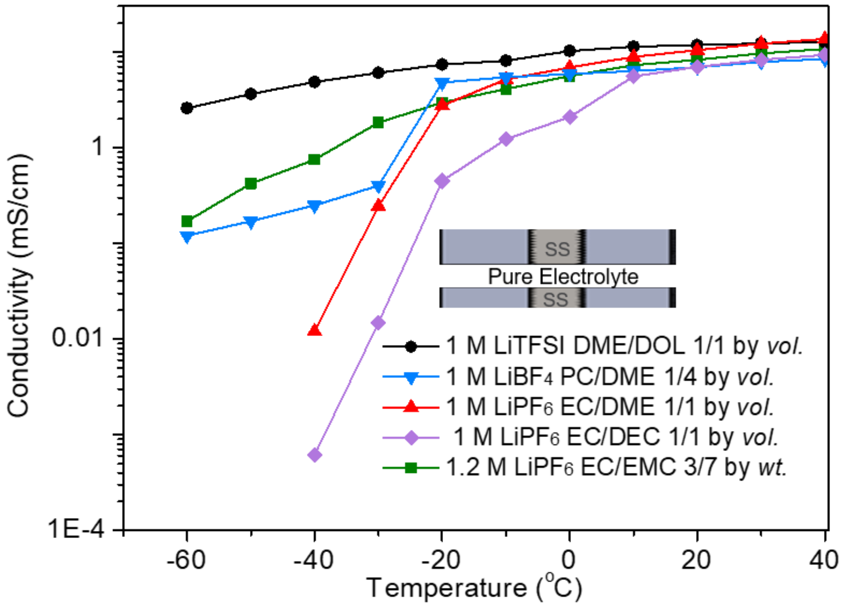

Supplementary Figure 18 | Ionic conductivity of pure electrolyte with conventional liquid electrolytes. It should be noted that conventional liquid carbonate electrolytes will be frozen at such low temperate (*e.g.*, < -30 °C) and render extremely low conductivity and high charge-transfer impedance. While ether-based electrolyte can maintain a decent conductivity, such as the 1 M LiTFSI in DOL/DME system, it poses an extremely increased charge-transfer impedance at subzero temperature, partially due to the large desolvation energy of the dilute ether electrolyte.[7,8] This will increase the overpotential when discharging at reduced temperature, thereby leading to poor Li/CF_x performance.

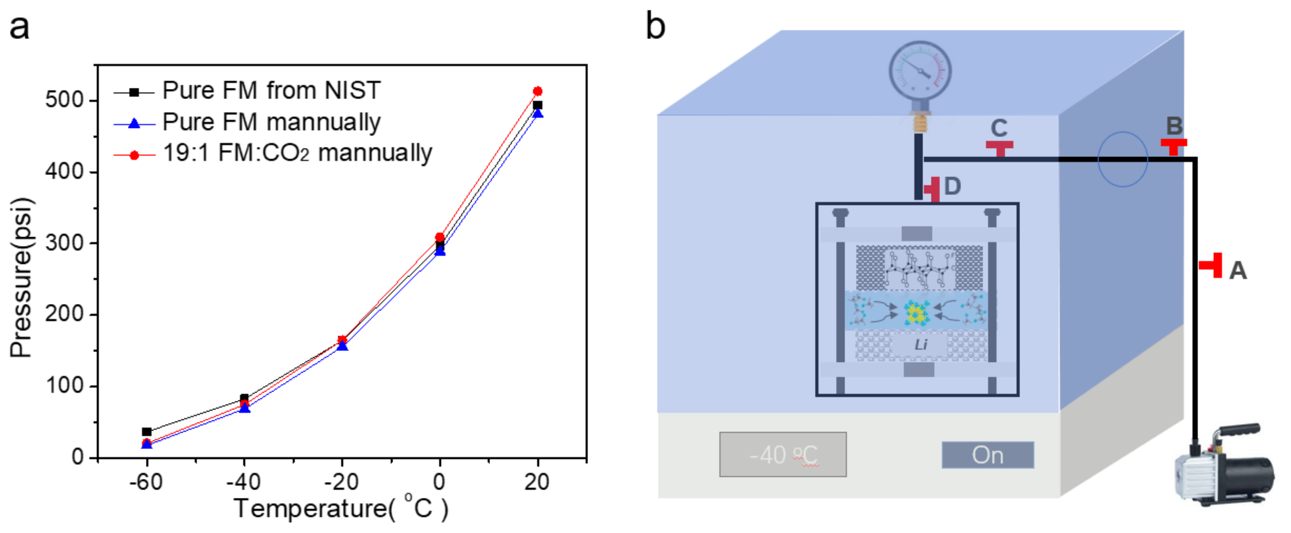

Supplementary Figure 19 | Pressure calibration. a, Pressure calibration of vapor pressure of FM and the mixture of FM and CO_2 at different temperatures. **b**, Schematic description of pressure tuning process. During test, valve D was kept open to record the pressure. By controlling the valves of A, B and C, the pressure inside the tested cell can be tuned to the set pressure.

Supplementary Table 2 | Quantification of ionic conductivity value comparison between MPM and commercial Celgard membranes at different pressures at -40 °C.

Pressure	MPM		Celgard	
	Bulk Resistance (Ohm)	Ionic Conductivity (mS/cm)	Bulk Resistance (Ohm)	Ionic Conductivity (mS/cm)
75 psi (*Pv*)	3173	0.113	5371	0.065
70 psi	15165	0.0217	192190	0.0015
65 psi	32908	0.009	1695000	0.0001

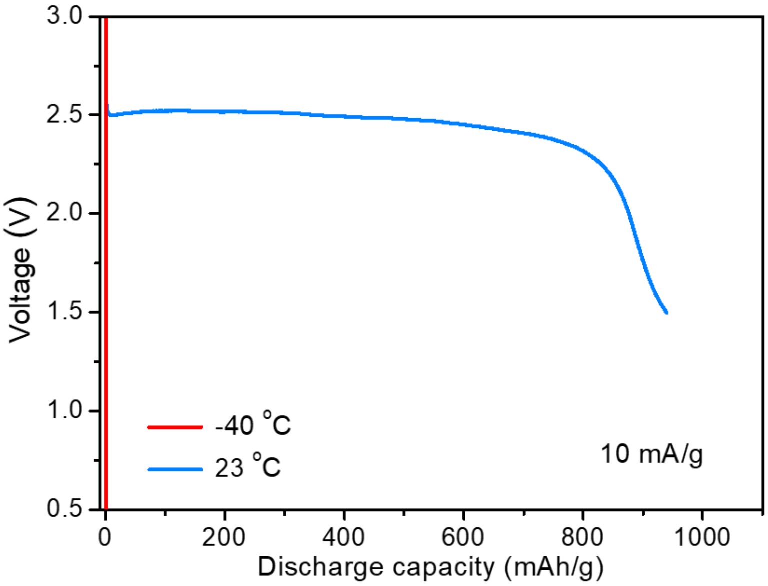

Supplementary Figure 20 | Discharge characteristics of Li//CF$_x$ cells with conventional liquid electrolyte system (1M LiPF$_6$ EC/DEC, 1:1 in volume) at room temperature and -40 ^{o}C.

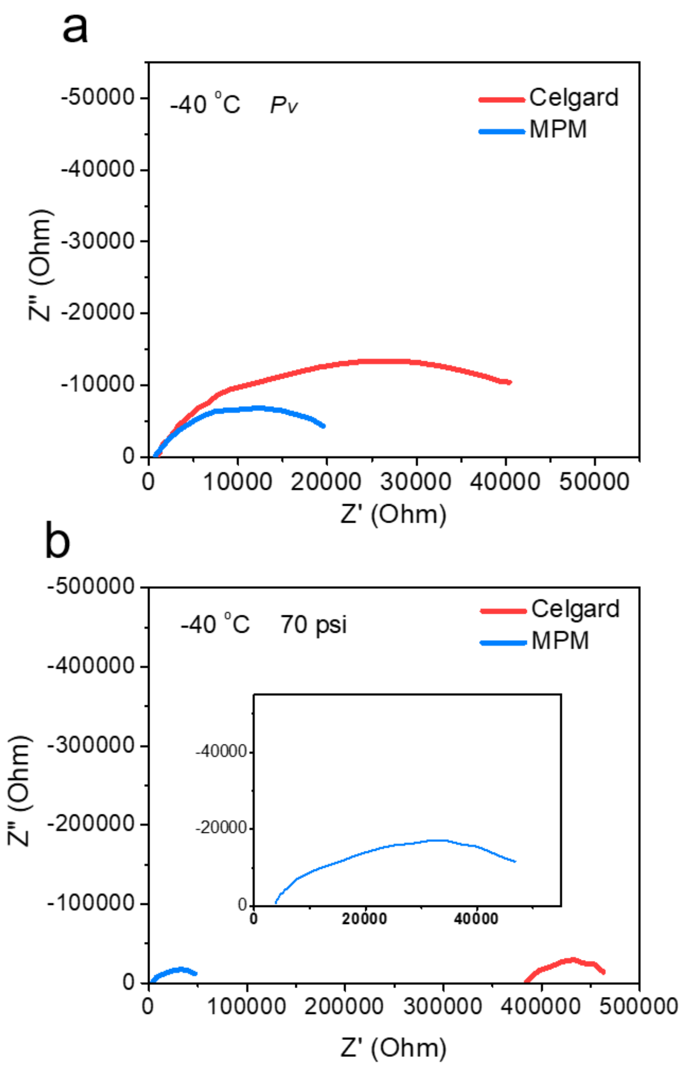

Supplementary Figure 21 | Nyquist impedance. Nyquist impedance of Li//CF_x cells mixed with 20 *wt*. % UiO-66 using Celgard and MPM at (**a**) vapor pressure and (**b**) 70 psi at -40 ºC.

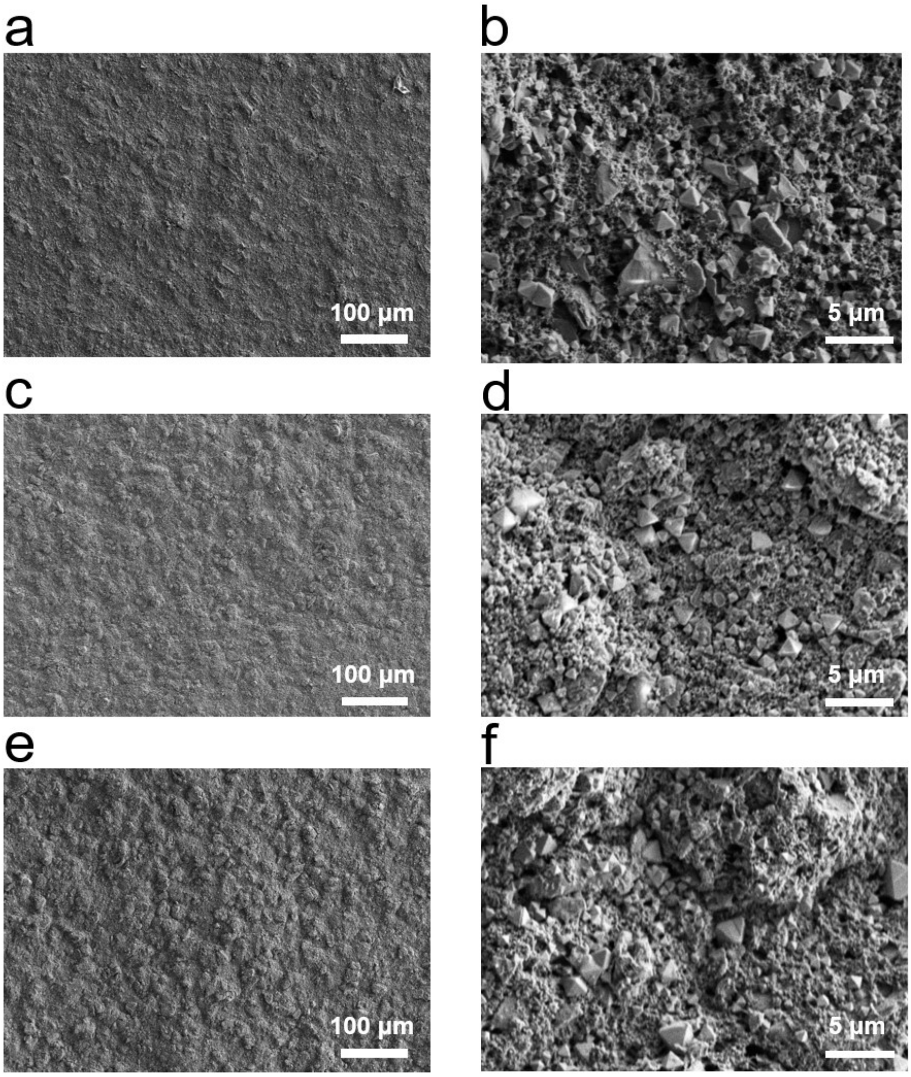

Supplementary Figure 22 | SEM images of CF_x electrodes with 20 wt% of UiO-66. a, **b** the pristine CF_x electrode; **c**, **d** the CF_x electrode after discharge at -40 °C and vapor pressure; **e**, **f** CF_x electrode after discharge at -40 °C and 70 psi.

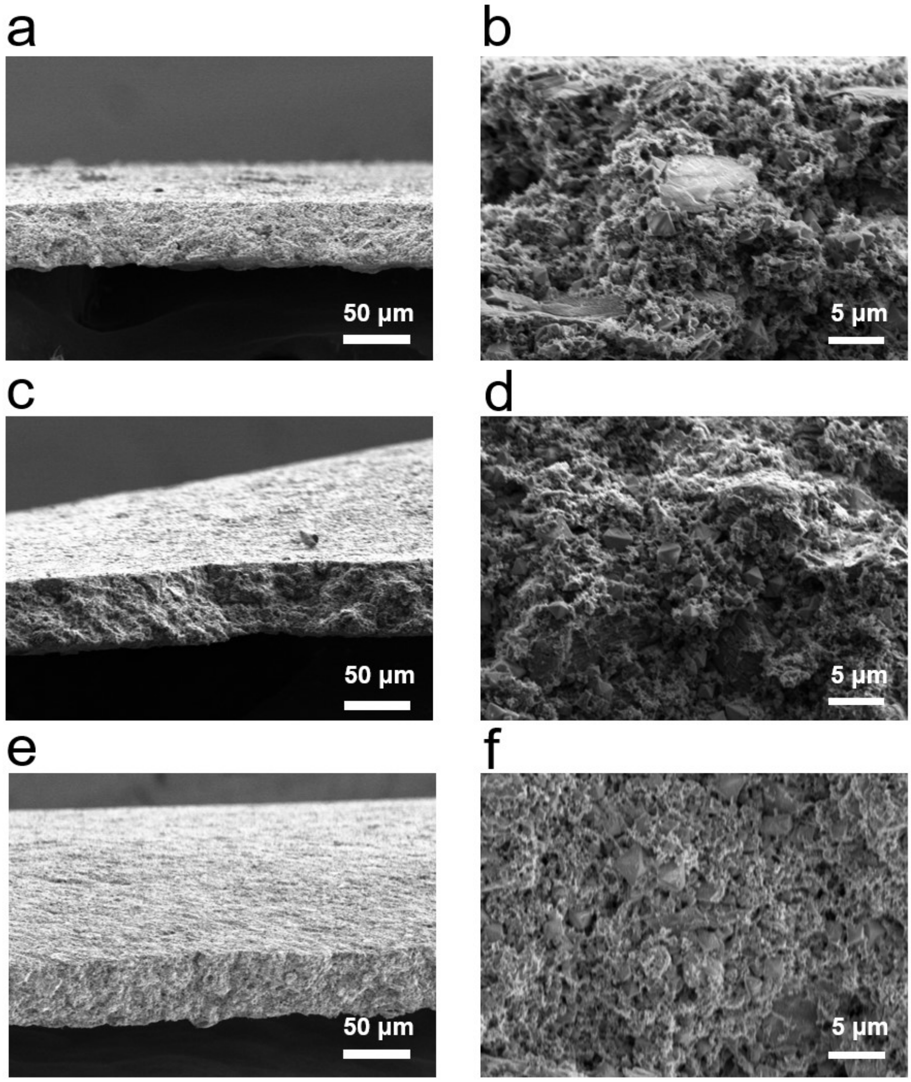

Supplementary Figure 23 | Cross-sectional SEM images of CF_x electrodes with 20 wt% of UiO-66. a, **b** the pristine electrode; **c**, **d** the electrode after discharge at -40 ºC and vapor pressure; **e**, **f** the electrode after discharge at -40 ºC and 70 psi.

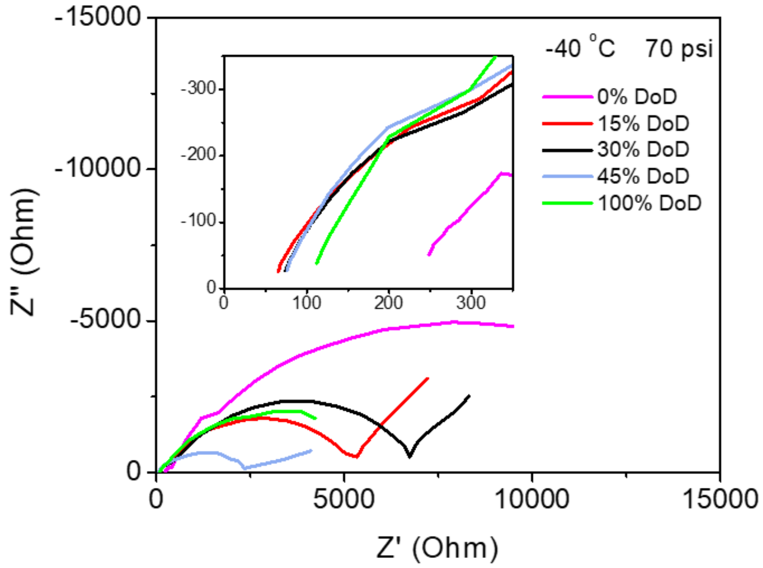

Supplementary Figure 24 | Nyquist impedance of Li//CF$_x$ cell (with 20 wt% of UiO-66 in the cathode) using MPM at 70 psi, -40 °C and different depths of discharge (DoDs). Inset shows the detailed comparison of bulk impedances at high frequency regions.

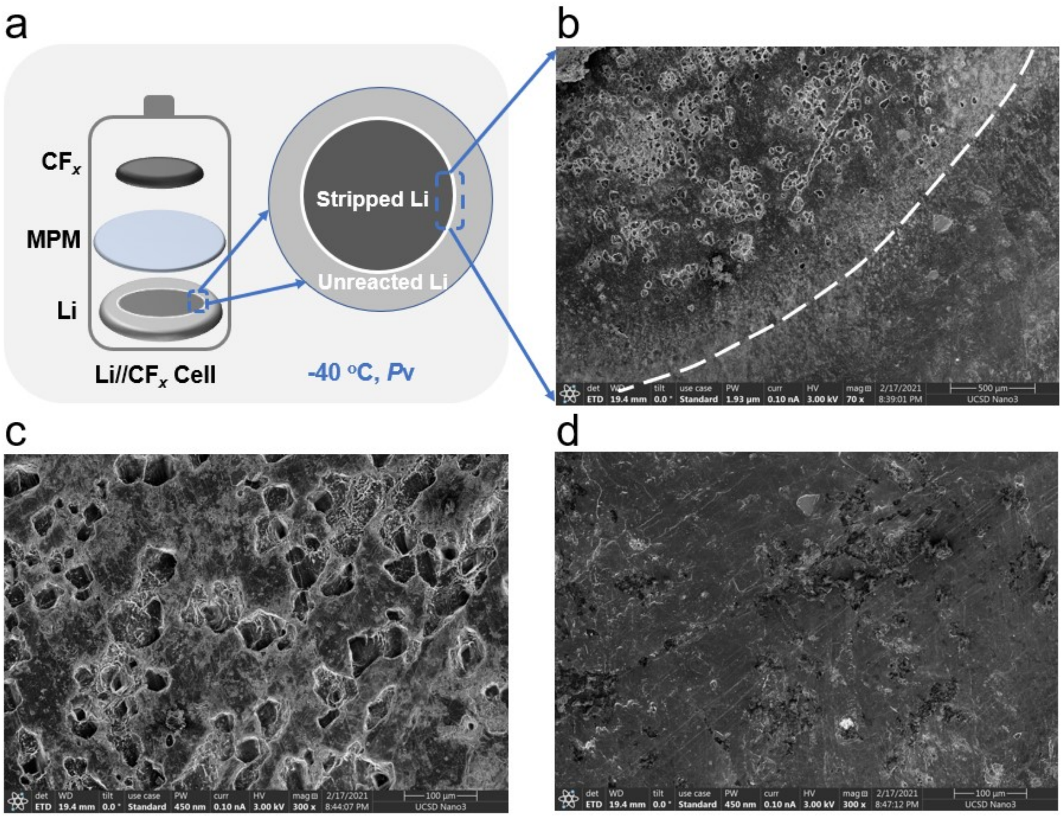

Supplementary Figure 25 | Characterization of stripped Li metal under vapor pressure (liquid state). a Schematic showing the of Li//CF_x cell with a relatively large Li chip as the anode while a small CF_x electrode disc as the cathode. **b** SEM image of the Li metal anode obtained from disassembling the Li//MPM//CF_x cell after discharging at -40 °C and vapor pressure. The white dotted line indicates the boundary between stripped and unreacted Li metal. The enlarged SEM images of (**c**) stripped and (**d**) unreacted Li metal.

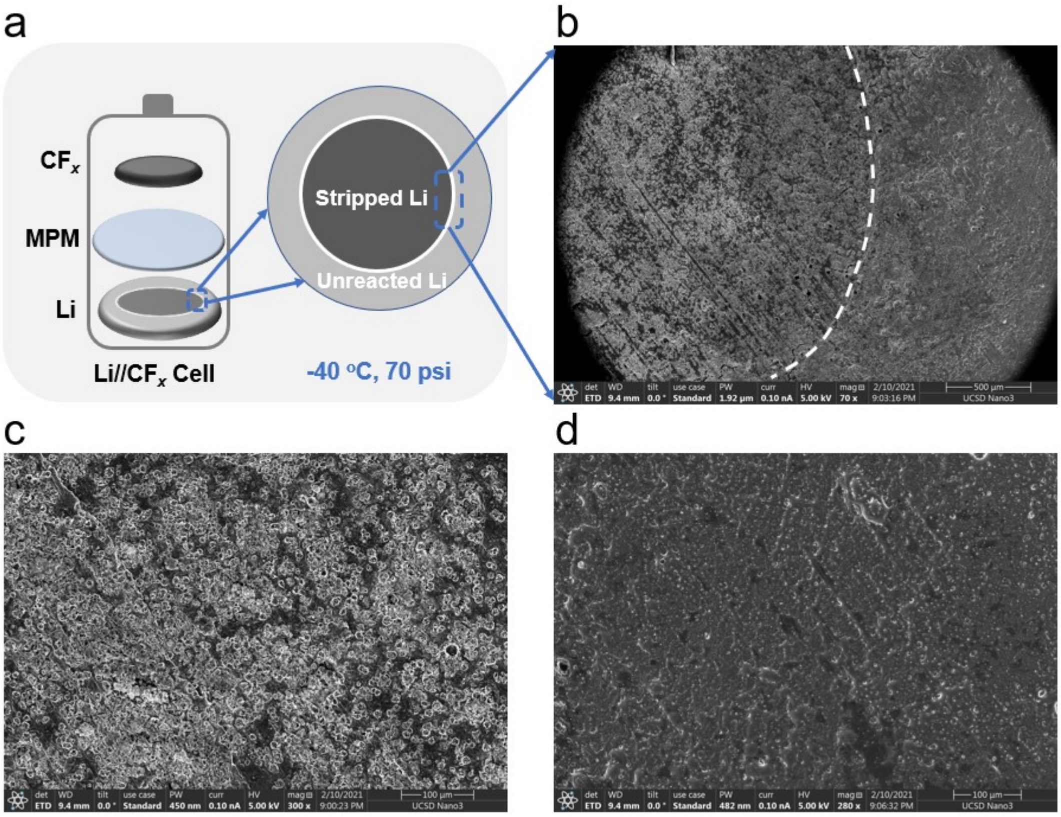

Supplementary Figure 26 | Characterization of stripped Li metal under reduce pressure. a Schematic showing the Li//CF$_x$ cell with a big Li chips as the anode while small CF$_x$ electrode disc as the cathode. **b** SEM image of Li metal achieved by disassembling the Li//MPM//CF$_x$ cell after discharging at -40 °C, and 70 psi. The white dotted line indicates the interface of stripped and unreacted Li metal. The enlarged SEM images of (**c**) stripped and (**d**) unreacted Li metal.

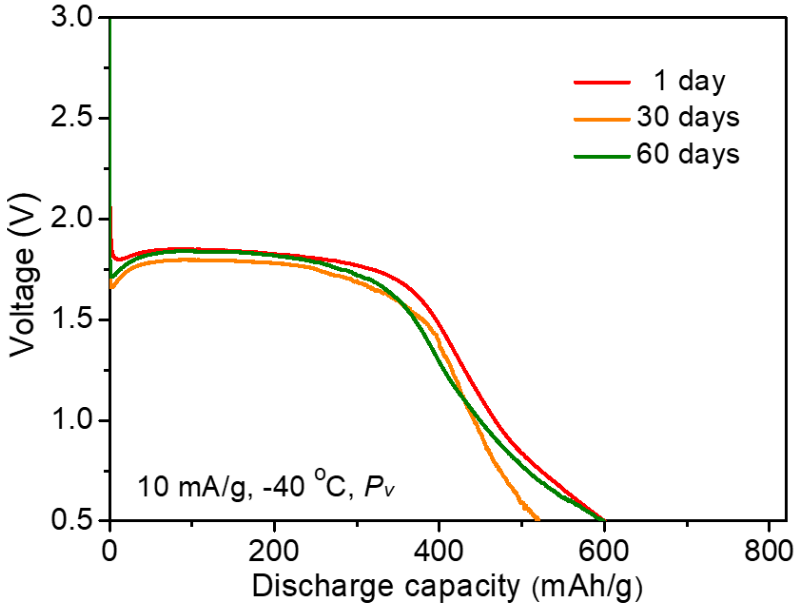

Supplementary Figure 27 | Self-discharge testing of Li//CF$_x$ cells with MPM confined LGEs. Three parallel cells were rested at room temperature and vapor pressure for 1, 30, and 60 days in sequence before discharging at -40 °C. Note that the slight variation of capacities between 1-, 30- and 60-days storage time might be due to the variations in cell assembly process including mass loadings, electrolyte/electrode thickness variations, gas feeding, and ohmic contact, which are often observed in home-made cells.

Supplementary References

1. Maia, J. M. & Mota, J. Molecular simulation of gas adsorption equilibria in nanoporous materials, (UNL Repository, 2014).
2. Boyd, P. G., Moosavi, S. M., Witman, M. & Smit B. Force-field prediction of materials in metal-organic frameworks, *J. Phys. Chem. Lett.* **8**, 357-363 (2017).
3. Böhm, H. J., Meissner, C. & Ahlrichs R. Molecular dynamics simulation of liquid CH_3F, CHF_3, CH_3Cl, CH_3CN, CO_2 and CS_2 with new pair potentials, *Mol. Phys.* **53**, 651-672 (1984).
4. Aimoli, C. G., Maginn, E. J. & Abreu, C. R. A. Force field comparison and the thermodynamic property calculation of supercritical CO_2 and CH_4 using molecular dynamics simulations, *Fluid Phase Equilib*. **368**, 80-90 (2014).
5. Cmarik, G. E., Kim, M., Cohen, S. M. &Walton, K. S. Tuning the adsorption properties of UiO-66 via ligand functionalization. *Langmuir* **28**, 15606-15613 (2012).
6. Cavka, J. H., Grande, C. A., Mondino, G. & Blom, R. High pressure adsorption of CO_2 and CH_4 on Zr-MOFs. *Ind. Eng. Chem. Res.* **53**, 15500-15507 (2014).
7. Li, Q. *et al.* Li^+-desolvation dictating lithium-ion battery's low-temperature performances. *ACS Appl. Mater. Interfaces* **9**, 42761-42768 (2017).
8. Borodin, O., Self, J., Persson, K.A., Wang, C. & Xu, K. Uncharted waters: super-concentrated electrolytes. *Joule* **4**, 69-100 (2020).
