# Supplementary material for: Sub-nanometer confinement enables facile condensation of gas electrolyte for low-temperature batteries
Source: Nat Commun. 2021 Jun 7;12:3395. doi: 10.1038/s41467-021-23603-0 (PMC8184934; doi:10.1038/s41467-021-23603-0)
Supplement: Supplementary file 2 — Description of Additional Supplementary Files [file 41467_2021_23603_MOESM2_ESM.docx]

**Description of Additional Supplementary Files**

File Name: Supplementary Movie 1

Description: Unfiltered Raw Data for the dynamic reconstruction of the MPM via Nano-CT.

File Name: Supplementary Movie 2

Description: Filtered and cropped data for the dynamic reconstruction of the MPM via Nano-CT.
